# A strategy to account for noise in the *X*-variable to reduce underestimation in Logan graphical analysis for quantifying receptor density in positron emission tomography

**DOI:** 10.1186/s12880-020-0421-6

**Published:** 2020-02-10

**Authors:** Paulus K. Shigwedha, Takahiro Yamada, Kohei Hanaoka, Kazunari Ishii, Yuichi Kimura, Yutaka Fukuoka

**Affiliations:** 1 Department of Electrical Engineering and Electronics, Graduate School of Engineering, Kogakuin University, Shinjuku, Tokyo, Japan; 2 Department of Computational Systems Biology, Faculty of Biology-Oriented Science and Technology, Kindai University, Kinokawa, Wakayama, Japan; 3 Institute of Advanced Clinical Medicine, Kindai University, Osakasayama, Osaka, Japan; 4 Department of Radiology, Faculty of Medicine, Kindai University, Osakasayama, Osaka, Japan

**Keywords:** Positron emission tomography, Logan graphical analysis, Receptor parametric imaging, Binding potential, LSC

## Abstract

**Background:**

The Logan graphical analysis (LGA) algorithm is widely used to quantify receptor density for parametric imaging in positron emission tomography (PET). Estimating receptor density, in terms of the non-displaceable binding potential (*BP*_*ND*_), from the LGA using the ordinary least-squares (OLS) method has been found to be negatively biased owing to noise in PET data. This is because OLS does not consider errors in the *X*-variable (predictor variable). Existing bias reduction methods can either only reduce the bias slightly or reduce the bias accompanied by increased variation in the estimates. In this study, we addressed the bias reduction problem by applying a different regression method.

**Methods:**

We employed least-squares cubic (LSC) linear regression, which accounts for errors in both variables as well as the correlation of these errors. Noise-free PET data were simulated, for ^11^C-carfentanil kinetics, with known *BP*_*ND*_ values. Statistical noise was added to these data and the *BP*_*ND*_s were re-estimated from the noisy data by three methods, conventional LGA, multilinear reference tissue model 2 (MRTM2), and LSC-based LGA; the results were compared. The three methods were also compared in terms of beta amyloid (A *β*) quantification of ^11^C-Pittsburgh compound B brain PET data for two patients with Alzheimer’s disease and differing A *β* depositions.

**Results:**

Amongst the three methods, for both synthetic and actual data, LSC was the least biased, followed by MRTM2, and then the conventional LGA, which was the most biased. Variations in the LSC estimates were smaller than those in the MRTM2 estimates. LSC also required a shorter computational time than MRTM2.

**Conclusions:**

The results suggest that LSC provides a better trade-off between the bias and variability than the other two methods. In particular, LSC performed better than MRTM2 in all aspects; bias, variability, and computational time. This makes LSC a promising method for *BP*_*ND*_ parametric imaging in PET studies.

## Background

This study aims to propose an algorithm to improve the accuracy in the quantification of receptor density for parametric imaging in positron emission tomography (PET). By applying parametric imaging methods such as Logan graphical analysis (LGA) and multilinear reference tissue models (MRTM) to PET data, we can acquire the receptor density in terms of the non-displaceable binding potential (*BP*_*ND*_) index [1]. LGA is a time-efficient and a computationally efficient algorithm applicable to reversibly binding receptors [2, 3]. It transforms data of PET tissue time-activity curves (tTACs) into a simple linear relationship, and *BP*_*ND*_ can then be estimated from the slope of this relationship using the ordinary least-squares (OLS) method.

*BP*_*ND*_ compares the concentration of the administered radiotracer in receptor-rich to receptor-free regions[1]. It can therefore be defined as the equilibrium ratio of specifically bound radiotracer (in receptor-rich region) to nondisplaceable radiotracer (in receptor-free region) [1]. Moreover, *BP*_*ND*_ is proportional to the density of binding sites [1, 2], which denotes the concentration of target receptors in the region of interest.

Despite the advantages of the LGA, the estimated *BP*_*ND*_ values have been found to be underestimated in cases of noisy PET data. This bias has been observed to increase with an increase in the magnitude of both *BP*_*ND*_ and noise [4–6]. Specifically, the bias is due to noise in the *X*-variable (predictor variable). The noise in the predictor variable is attributed to the noisy term, *C*(*t*) (see Eq. (1)), which is the denominator in both the LGA variables [6, 7]. Accordingly, the noise due to *C*(*t*) propagates correlatively in the LGA variables, presenting a correlated errors-in-variables (EIV) problem. Thus, both the predictor and response variables of the LGA are contaminated with correlated noise, yet the OLS method only accounts for the errors in the response variable. This explicitly makes OLS unsuitable for estimating the *BP*_*ND*_.

A variety of methods have been proposed to reduce the bias in *BP*_*ND*_ estimates. These methods range from tTAC smoothing methods, based on the kinetics of an administered radiotracer, such as the generalized linear least-squares (GLLS) [8] technique, to methods based on a rearrangement of the original LGA equation, such as the multilinear reference tissue models (MRTM 1 & 2) [7, 9]. Other methods including orthogonal distance regression (ODR) [10] simply employ a different line estimation technique. These methods, however, have been found to either only slightly reduce the bias or reduce the bias at the cost of worsened estimation deviation [4, 5]. This necessitates the need for an improved method.

It has been shown that the regression method used to estimate the Logan slope influences the resulting bias in *BP*_*ND*_ estimates [6, 10]. On this basis, the present study employs an alternative linear regression method referred to as least-squares cubic (LSC) [11–13], which fully accounts for errors in both variables, to estimate *BP*_*ND*_. LSC minimizes the squared residuals in both the predictor and response variables and incorporates the correlation of errors in these variables [12, 13]. Thus, LSC is expected to be appropriate for correlated errors in LGA variables. Furthermore, LSC has been shown to improve regression parameter estimates in the fields of biogeosciences [14] and geophysics [13].

To the best of our knowledge, LSC regression (as in [12, 13]) has not been applied to the LGA. In this study, LSC was applied to the LGA in an effort to reduce bias in the estimates of *BP*_*ND*_. With the OLS-based LGA as the conventional method, the viability of LSC in reducing bias in *BP*_*ND*_ estimates was assessed against MRTM2, which is currently a widely accepted method.

The three methods, LSC, MRTM2 and the conventional LGA, were compared using a well known radiotracer, ^11^C-carfentanil (CFN), for simulation studies. To account for differing binding characteristics, for human data studies, the three methods were verified using a different and relatively new radiotracer, ^11^C-Pittsburgh compound B (^11^C-PIB), which is gaining popularity owing to its usage in the Alzheimer’s Disease Neuroimaging Initiative (ADNI) studies. Both CFN and ^11^C-PIB have specific binding sites in the brain. CFN binds to *μ*-opioid receptors, and ^11^C-PIB binds to beta amyloid (A *β*) plaques.

## Methods

### Logan graphical analysis

LGA is a well-established algorithm for estimating the density of specific binding sites of receptors in terms of the *BP*_*ND*_ [1]. The operational equation of the LGA is as given as [2],

1 $$\begin{array}{*{20}l}  &\frac{\int_{0}^{t}C(u)\,du}{C(t)} = (1 + BP_{ND}) \\ &\:\:\:\:\:\:\:\:\:\:\: \cdot\left(\frac{\int_{0}^{t}C^{R}(u)\,du + \frac{1}{k^{R}_{2}}C^{R}(t)}{C(t)}\right) - \text{int}. \end{array} $$

*C*(*t*) and *C*^*R*^(*t*) represent the total radioactivity concentration in a brain tissue and a reference region, respectively. A reference region is such that it has ignorable specific binding sites and a density of nonspecific binding sites equal to that of the other brain tissues. It is defined by the biochemical aspects of the administered radiotracer, and in terms of amyloid imaging, cerebellum gray matter is used as the reference region [15].

Equation (1) is such that after some time *t*^∗^, the *y*-intercept term, int, becomes constant with time, and thus, Eq. (1) becomes a linear relationship between ${\frac {\int _{0}^{t}C(u)du}{C(t)}}$ and ${\frac {\int _{0}^{t}C^{R}(u)\,du + \frac {1}{k^{R}_{2}} C^{R}(t)}{C(t)}}$, with a slope of, 1+*BP*_*ND*_.

We can then apply a linear regression to Eq. (1), for *T*>*t*^∗^, to compute *BP*_*ND*_ from its slope. A linear regression is fast and stable, and therefore, it is suitable for application in a voxel-by-voxel fashion to construct a *BP*_*ND*_ parametric image.

**Fig. 1:**
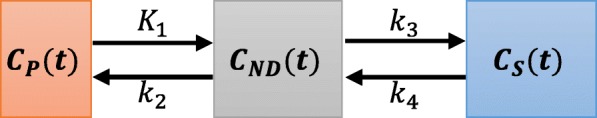
Two-tissue compartmental model. Transportation of the administered radiotracer between the compartments, *C*_*P*_,*C*_*ND*_ and *C*_*S*_ is described by the rate constants, *K*_1_, *k*_2–4_

Figure 1 shows a compartmental model used to describe the kinetics of a radiotracer in brain tissues [16]. The compartments *C*_*P*_, *C*_*ND*_ and *C*_*S*_ represent the concentration of the radiotracer in arterial plasma, non-displaceable (bound-free + non-specifically bound) radiotracer, and specifically bound radiotracer, respectively. The transportation of the radiotracer between neighboring compartments is described by the rate constants, *K*_1_ [*mlg*^−1^*min*^−1^] and *k*_2–4_ [*min*^−1^]. *K*_1_ denotes the delivery rate of the radiotracer from the arterial plasma to tissues. *k*_2_ is the clearance rate from the tissues back to the plasma, and *k*_3_ and *k*_4_ are the respective association and dissociation rates of the radiotracer to and from the specific binding sites. More details on the compartmental model can be found in [16].

MRTM1 reduces the noise by rearranging the original LGA equation such that the noisy term, *C*(*t*), only appears on the side of the response variable, thus allowing OLS to account for all errors in the LGA variables due to *C*(*t*) [9]. MRTM1 results in multilinear regression, with three regression parameters that have to be estimated. To further stabilize MRTM1, MRTM2 was developed by reducing the number of regression parameters from three to two [9]. *BP*_*ND*_ can then be estimated from the regression parameters obtained by MRTM2.

### Least-squares cubic

LSC is an EIV-based regression method. LSC fully considers the errors in both variables, by minimizing the sum of weighted squared errors in both the involved variables, and by including the correlation of these errors [11–13]. Under specific assumptions, LSC can be shown to reduce to either OLS or other common regression methods, rendering LSC to be a general solution to the linear regression problem [11]. Here, we introduce LSC regression and demonstrate how it can be used to estimate *BP*_*ND*_ from LGA.

To fit a straight line model, *y*=*αx*+*β*, LSC [12] estimates the regression parameters by minimizing the equation,

2 $$\begin{array}{*{20}l}  \sum \limits_{i=1}^{N}d^{2}_{i} &= \sum \limits_{i=1}^{N} \left\{ w(X_{i})(x'_{i} -X_{i})^{2} \right. \\ & - 2r\sqrt{w(X_{i})w(Y_{i})}(x'_{i} - X_{i})(y'_{i} -Y_{i}) \\ & \left. + w(Y_{i})(y'_{i} - Y_{i})^{2} \right\}\frac{1}{1-r^{2}}, \end{array} $$

where *α* and *β* are the slope and *y*-intercept of the fitted straight line, respectively. (*w*(*X*_*i*_),*w*(*Y*_*i*_)) is a pair of weights for the corresponding pair of observed data, {(*X*_*i*_,*Y*_*i*_) | *i*=1,2,...,*N*}, and *r* is the correlation of the errors in the observed data. (*xi*′,*yi*′) is a pair of the estimates of the true points (*x*_*i*_,*y*_*i*_).

It can then be seen from Eq. (2) that LSC accounts for the weights (*w*(*X*_*i*_), *w*(*Y*_*i*_)) and errors (*xi*′−*X*_*i*_, *yi*′−*Y*_*i*_) in both variables, as well as the correlation of these errors (*r*). Accordingly, a regression method that does not consider either of these aspects makes assumptions about them in certain ways. For example, an unweighted method assumes equal weights of ones; minimizing only the residuals of the response variable means assuming that the predictor variable is error-free and not considering the correlation of errors means assuming that the errors in the measured data are uncorrelated.

Solving Eq. (2) for the slope (*α*^′^) and *y*-intercept (*β*^′^) gives,

3 $$ \alpha' = \frac{\sum \limits_{i=1}^{N}W_{i} B_{i} V_{i}}{\sum \limits_{i=1}^{N}W_{i} B_{i} U_{i}} \:\:\:\:\:\:\:\:\: \text{and}\:\:\:\:\:\:\:\:\: \beta' = \overline{Y} - \alpha'\overline{X},  $$

where,

$$  U_{i} = X_{i} - \overline{X} \:\:\:\:\:\:\:\:\: \text{and}\:\:\:\:\:\:\:\:\: V_{i} = Y_{i} - \overline{Y}   $$

4 $$\begin{array}{*{20}l}  W_{i} &= \frac{w(X_{i})w(Y_{i})}{\alpha^{'2}w(Y_{i}) + w(X_{i}) - 2\alpha'r\sqrt{w(X_{i})w(Y_{i})}}, \\ B_{i} &= W_{i} \left(\frac{U_{i}}{w(Y_{i})} + \frac{\alpha' V_{i}}{w(X_{i})} \right.  \\ & \left. - (\alpha' U_{i} + V_{i})\frac{r}{\sqrt{w(X_{i})w(Y_{i})}}\right). \end{array} $$

${\overline {X} \;\; \text {and} \;\; \overline {Y}}$ are given by,

5 $$  \overline{X} = \sum \limits_{i=1}^{N}W_{i} X_{i}\text{\(/\)}\sum \limits_{i=1}^{N}W_{i}; \;\;\; \overline{Y} = \sum \limits_{i=1}^{N}W_{i} Y_{i}\text{\(/\)}\sum \limits_{i=1}^{N}W_{i}.  $$

A step-by-step derivation of Eqs. (3) and (4) can be found in previous papers [11–13]. A more thorough derivation, including one of Eq. (2), is demonstrated in [17].

The expression for *α*^′^ in Eq. (3) includes *α*^′^, which is contained in *W*_*i*_ and *B*_*i*_ as shown in Eq. (4). This means that LSC requires an initial guess of *α*^′^; that is, it is an iterative procedure. The slope obtained by OLS can be used as the initial guess of *α*^′^. Given the PET data, *C*(*t*) and *C*^*R*^(*t*), LSC can be used to estimate *BP*_*ND*_ by the slope in Eq. (3).

#### Weights, { *w*(*X*_*i*_), *w*(*Y*_*i*_)}, and correlation of errors in variables (*r*)

For LGA, the weights, *w*(*X*_*i*_) and *w*(*Y*_*i*_), and the correlation of errors, *r*, are unknown; therefore, they have to be estimated. To this end, we adopted the method of "relative error regression" [18, 19]. In relative error regression, instead of minimizing the squared-sum of the absolute errors, ${\sum (y^{\prime }_{i} - Y_{i})^{2}}$, the squared-sum of the relative errors, ${\sum [(y^{\prime }_{i} - Y_{i})/Y_{i}]^{2}}$, is minimized. In statistical prediction, relative errors are considered to be more informative than absolute errors [19]. Expressing the relative errors in this manner can help adjust measurements in a weighted linear regression with the weights given by, ${1/Y_{i}^{2}}$ [18, 19]. After adopting this technique for LSC, the *X* and *Y* weights can be respectively given as,

6 $$  w(X_{i}) = 1/X_{i}^{2} \:\:\:\:\:\: \text{and}\:\:\:\:\:\: w(Y_{i}) = 1/Y_{i}^{2}.  $$

A detailed analysis demonstrating the viability of these weight functions for LSC-based LGA is given in Additional file 1.

The errors in the variables were unknown; thus, their correlations were estimated by the correlations between the variables themselves. These correlations were estimated by the “correlations of determination." The correlation of determination is a number between 0 and 1, and it determines the proportion of variation in the response variable that can be explained by the predictor variable [20, 21]. To iterate, the slope obtained in the first run was substituted in *W*_*i*_ and *B*_*i*_ in Eq. (4). The iteration process was repeated until the relative differences between consecutive estimates of the slope, (*αi*′−*αi*+1′)/*αi*+1′, were less than 10^−10^. A thorough step-by-step guide for using LSC can be found in [13].

### Simulation studies

In order to assess the performance of LSC against that of MRTM2 and conventional LGA when estimating *BP*_*ND*_, a set of PET data of the two-tissue compartmental model was simulated using a clinically measured plasma time-activity curve in a PET study. The kinetic parameters, *K*_1_,*k*_2–4_ and the non-displaceable distribution volume (*V*_*ND*_) used for simulations were adopted from the range of values used in [22], mimicking CFN;

- 11 values of *BP*_*ND*_ were set in the range of [0.0, 3.0], covering a range of [0.0, 0.35] for *k*_3_.
- [*K*_1_*k*_4_]=[0.1835 mL cm^−1^ min^−1^ 0.115/min].
- [*k*_2_*k*_3_]=[*K*_1_/*V*_*ND*_*BP*_*ND*_·*k*_4_].
- *V*_*ND*_ =1.59 mL cm^−1^.

The reference region was formed with a zero noise level, zero *BP*_*ND*_, and delivery and clearance rates equal to those of the target tissues, due to its physiological assumptions. Using these kinetic parameter values, 11 noise-free tTACs corresponding to the 11 *BP*_*ND*_ values were formed. Statistical noise was added to these noise-free tTACs, and 1024 noisy tTACs were simulated as a slice of 32-by-32 pixels for each noise-free tTAC. The *BP*_*ND*_ values were then re-estimated from the noisy tTACs by LSC, MRTM2, and conventional LGA, and the results were compared. The true value for the dissociation rate of the reference region (${k^{R}_{2}}$), which was used to produce the simulation data, was used for all methods.

**Fig. 2:**
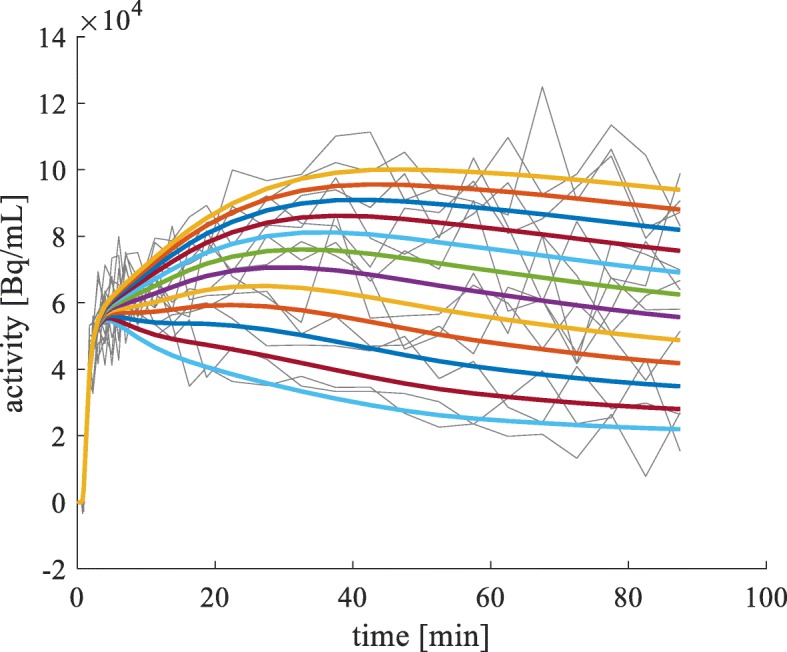
Simulated tTACs. The 12 thick curves denote the noise-free tTACs. Eleven of these correspond to the 11 *BP*_*ND*_ values. The remaining one (lowermost) represents the reference region. The thin curves denote the noisy tTACs. Only one noisy tTAC is shown out of the 1024 simulated noisy tTACs for each of the noise-free tTACs

The noise was assumed to have a zero-mean Gaussian distribution with a variance proportional to the true tTAC; this is the same as the one used in previous studies [4, 8]. The noise scaling factor was set such that the actual magnitude of the noise level in the simulated tTACs varied within a range of 0 to 30%; this range covered the actual noise observed in voxel-based PET data. The magnitudes of the noise level were calculated by the percentage of the ratios of the “standard deviation of the noisy tTACs" to the “mean of the noise-free tTAC" for the portion of the time range used for the *BP*_*ND*_ estimations. Figure 2 shows the simulated noise-free (thick lines) and their corresponding noisy (thin lines) tTACs for the 11 *BP*_*ND*_ values. The 12th blue curve at the bottom is the curve of the reference region.

### Human data studies

Two patients, of which one is A *β*-negative and the other is A *β*-positive, participated in this study. The two patients underwent a ^11^C-PIB PET scan for 70 minutes. The scan was carried out according to the standard ADNI ^11^C-PIB PET procedure. The study protocol was approved by the Ethics Committee of Kindai University Hospital, and written informed consent was obtained from the participants.

The data were collected in matrices of 128 × 128 × 47 slices, and voxel sizes of 2.1 × 2.1 × 3.4 mm^3^. *BP*_*ND*_ parametric images – reflecting A *β* deposits in the brain regions – were then generated using the three algorithms, LSC, MRTM2, and conventional LGA, implemented in MATLAB (The MathWorks, Inc., Natick, MA, United States).

Time *t*^∗^(30 min.), which was used for both synthetic and actual data, was graphically determined from simulated data, as demonstrated in Additional file 2.

For actual data, the values of ${k^{R}_{2}}$ that were used for all methods were estimated by MRTM1. MRTM1 allows estimation of ${k^{R}_{2}}$ with little bias [9]. Specifically for each slice, the average value, ${\bar {k}^{R}_{2}}$, calculated from the ${ \mid k^{R}_{2} \mid }$ of all voxels in the reference region, was used.

## Results

### Simulation studies

**Fig. 3:**
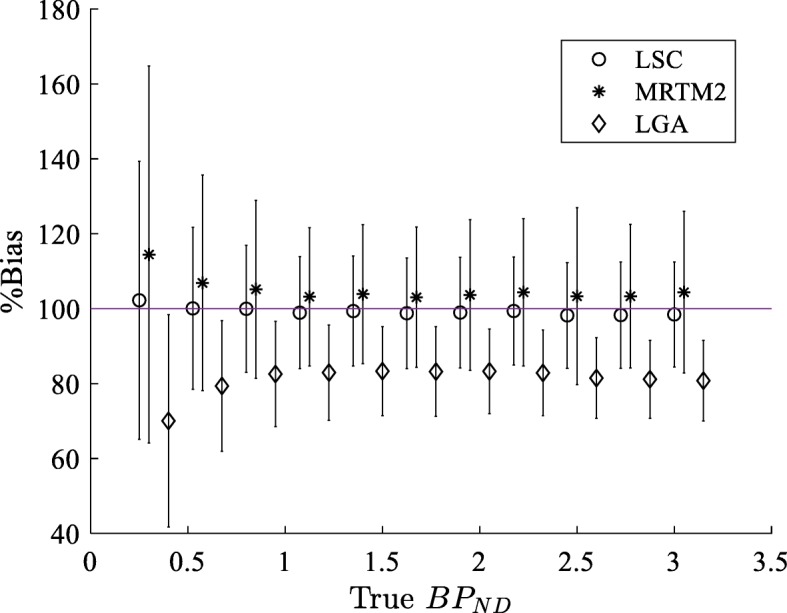
Comparisons of the percentage bias and variability in the estimated *BP*_*ND*_ values. The percentage bias and variability in the *BP*_*ND*_ estimated from the noisy tTACs by LSC, MRTM2, and the conventional LGA, are compared for the 11 values of true *BP*_*ND*_

The results in Fig. 3 compares the percentage bias in the *BP*_*ND*_ values estimated by the three methods from the noisy tTACs. The standard deviations (in percentages) are shown as error bars.

In these results, LSC estimates are the least biased along the entire range of *BP*_*ND*_, with up to a maximum of 12% and 28% bias difference in comparison with MRTM2 and conventional LGA, respectively. The error bars show that while the conventional LGA estimates have the smallest variability, LSC estimates have smaller variability than MRTM2 estimates.

### Human data studies

**Fig. 4:**
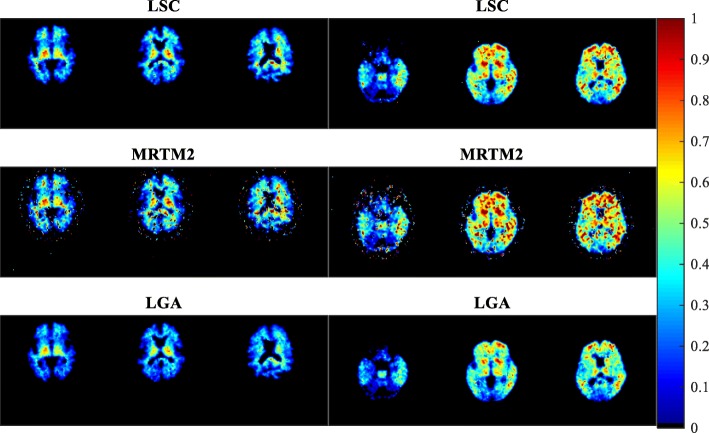
Axial slice images of brain regions obtained by the three methods, LSC, MRTM2 and conventional LGA. These are *BP*_*ND*_ images of A *β* quantification of ^11^C-PIB brain PET data of the two patients. The left panel shows the images of the A *β*-negative patient, and the right panel shows the images of the A *β*-positive patient

Figure 4 shows the *BP*_*ND*_ parametric images obtained by LSC, MRTM2, and conventional LGA. These images are generated from ^11^C-PIB PET brain images, and therefore they reflect A *β* deposits in the brain regions. This is because A *β* is the target receptor for ^11^C-PIB radiotracer. The left panel shows images of three slices of the A *β*-negative patient, obtained by each of the three methods, whereas the right panel shows the images of the A *β*-positive patient.

The images obtained by LSC have significantly higher total *BP*_*ND*_ estimates than those of conventional LGA for both patients. The images obtained by MRTM2 have the highest total *BP*_*ND*_ estimates. For MRTM2 images, however, considerably higher levels of noise are observed. These results are consistent with those of CFN observed in the simulated data.

Table 1 shows the average computational time taken by each method for the corresponding sets of three images in Fig. 4. LSC took moderate computational time, one order of magnitude higher than the conventional LGA, for both patients’ images. This was expected of LSC because it is an iterative procedure. MRTM2 took the longest computational time amongst the three methods, two orders of magnitude higher than the conventional LGA, for both patients’ images.

**Table 1:** Means (± standard deviations) of computational times taken by each method for the respective slice images in Fig. 4

	Human data computational time (seconds)	
	L	R
**LSC**	0.161 ±0.033	0.156 ±0.016
**MRTM2**	1.045 ±0.031	1.014 ±0.031
**LGA**	0.073 ±0.024	0.083 ±0.009

The two columns indicated by L and R denote the left and right panel images, respectively

## Discussion

In this study, LSC estimates of the *BP*_*ND*_ emerged to be less biased than those of MRTM2 and conventional LGA. Moreover, LSC estimates have smaller variations in comparison with those for MRTM2, providing a better trade-off between bias and variability.

The underestimation which is observed in the simulated data for the conventional LGA estimates is also reflected in the real data, and accordingly, the reduction of the underestimation seen in the simulated data for the LSC method is also reflected in the real data. Furthermore, the underestimations observed for the conventional LGA method in both simulation and human data studies are in consistency with previous studies [15, 22], and this strengthens the reliability of the results reported here.

Both LSC and OLS are based on the assumption that the errors in the measurements are normally distributed. However, this assumption may not always be satisfied. A visual inspection of the distributions of the LSC error values and the OLS residuals showed that the LSC error values have a better normal distribution compared with that of the OLS residuals (Additional file 3). This can be attributed to the fact that LSC takes into account the varying magnitude of errors at each data point as demonstrated in [12].

LSC considers the errors in both variables without altering the original LGA equation, thus maintaining its simplicity and inducing less variability. In addition, LSC does not only consider the errors associated with term *C*(*t*), but also the errors associated with each LGA variable as a whole. The effort to include more details in LSC has increased the number of parameters in Eq. (2), compared with the fundamental OLS regression, which only comprises the residuals of the response variable. This could be a factor in the slight increase in the variability of LSC estimates, compared with the conventional LGA estimates.

MRTM2 rearranges the conventional LGA equation into a multilinear regression form so that the noted noisy term, *C*(*t*), only appears in the response variable [7, 9]. This only considers the errors associated with *C*(*t*). However, the rest of the terms in the LGA equation are not entirely noise-free; they are less noisy compared with *C*(*t*). Not considering the noise in the rest of the terms could lead to the bias in the MRTM2 estimates.

Based on the computational times of the three methods for obtaining slice images, the conventional LGA has the highest computational efficiency, followed by LSC, and MRTM2. The computational time for LSC would depend on the number of iterations, which in turn depends on the iteration condition, with the computational time increasing with the number of iterations. All LSC images in Fig. 4 required at most three iterations. The iteration limiting condition in this study, (*αi*′−*αi*+1′)/*αi*+1′<10^−10^, was arbitrarily chosen. However, it was found that by choosing a stricter condition, say 10^−11^, the number of iterations did not increase.

Although the weighting formulas in Eq. (6) are inverse squares of the measurements, they will not cause the usual problem of overproportional weighting of smaller measurements. This is because they are not the direct weighting coefficients. The final weighting coefficients are appearing in Eq. (3) as *W*_*i*_*B*_*i*_. Because they appear in both the numerator and denominator, the overprortional weighting effect can be canceled out. Based on an analysis of LSC regression carried out in [12], it was shown that a set of weight functions satisfying the relation, ${\sqrt {\frac {w(X_{i})}{w(Y_{i})}} = \frac {\int _{0}^{t_{i}} C_{T}(u)\,du}{\int _{0}^{t_{i}}C^{R}(u)\,du + C^{R}(t_{i})/ k^{R}_{2}}}$, would be appropriate weights for the LSC-based LGA. The weighting formulas given in Eq. (6) satisfy this condition, therefore making them appropriate (See Additional file 1 for details).

## Conclusions

The proposed method, LSC-based LGA, significantly reduced the bias in the estimates of *BP*_*ND*_, up to 12% and 28% difference compared with MRTM2 and the conventional LGA, respectively. Considering the conventional LGA as the standard method, LSC reduced the bias at a slight expense of increased variation. However, LSC caused smaller variations and required shorter computational times than MRTM2. Thus, LSC outperformed MRTM2 in all aspects (bias, variability, and computational efficiency), making it a promising tool for the *BP*_*ND*_ estimation. These results are consitent as such for the two radiotracers respectively used for simulated and human data studies.

This study investigated the performance of LSC-based LGA mostly based on simulations, and thus, the method must be validated using a larger cohort of actual data. In addition, different weighting techniques may perform differently. Therefore, a further study is required to seek an adequate weighting technique for LGA.

## Supplementary information

**Additional file 1** Mathematical analysis of the LSC regression method. This analysis demonstrates the appropriateness of the weight functions used in this study.

**Additional file 2** tTACs and the corresponding Logan plot. The upper panel shows the mean tTACs of the region of interest (*BP*_*ND*_=3.00) and the reference region. The lower panel shows the corresponding Logan plot. By visual observation of where the data points in the Logan plot attain a linear relationship, the point corresponding to *Time*=30 minutes was chosen; hence, *t*^∗^=30 min was used in this study. The data points (in the lower panel) used for *BP*_*ND*_ estimation are dotted in the middle.

**Additional file 3** Comparisons of the distributions of the errors. This figure shows the distributions of the errors estimated by LSC (upper panel) and those estimated by OLS (lower panel).

## Data Availability

The data used and analyzed during the current study are available on reasonable request.

## Abbreviations

A *β*: Beta amyloid
ADNI: Alzheimer’s Disease Neuroimaging Initiative
*B**P*_*ND*_: Non-displaceable binding potential
CFN: ^11^C-carfentanil
*C*_*ND*_: Non-displaceable radiotracer
*C*_*P*_: Radiotracer in plasma
*C*_*S*_: Specifically bound radiotracer
^11^C-PIB: ^11^C-Pittsburgh compound B
EIV: Errors-in-variables
GLLS: Generalised linear least-squares
LGA: Logan graphical analysis
LSC: Least-squares cubic
MRTM: Multilinear reference tissue model
MRTM1: Multilinear reference tissue model 1
MRTM2: Multilinear reference tissue model 2
ODR: Orthogonal distance regression
OLS: Ordinary least-squares
PET: Positron emission tomography
tTACs: Tissue time-activity curves
*V*_*ND*_: Non-displaceable distribution volume
