# Supplementary material for: A strategy to account for noise in the X-variable to reduce underestimation in Logan graphical analysis for quantifying receptor density in positron emission tomography
Source: BMC Med Imaging. 2020 Feb 10;20:15. doi: 10.1186/s12880-020-0421-6 (PMC7011280; doi:10.1186/s12880-020-0421-6)
Supplement: Supplementary file 1 — Additional file 1 Mathematical analysis of the LSC regression method. This analysis demonstrates the appropriateness of the weight functions used in this study. [file 12880_2020_421_MOESM1_ESM.pdf]

Appendix

Let all variables be as defined in the main text. According to LSC [11], the respective residuals of the X and Y variables are given by,

$$x'_i - X_i = \frac{W_i(\beta' + \alpha' X_i - Y_i)(c_i - \alpha' w(Y_i)}{w(X_i)w(Y_i)} \quad \text{(A1)}$$

and

$$y'_i - Y_i = \frac{W_i(\beta' + \alpha' X_i - Y_i)(w(X_i) - \alpha' c_i)}{w(X_i)w(Y_i)}, \quad \text{(A2)}$$

where $c_i = r\sqrt{w(X_i)w(Y_i)}$.

Fig. S1 shows a schematic diagram for LSC. d_i denotes the residual between the observed point, $\boldsymbol{H}$, and the estimated point, $\boldsymbol{H}^*$. Based on a demonstration shown in [11], here the LSC is demonstrated specifically in terms of the LGA variables. Lets consider that the numerator components of the LGA variables, $\int_0^t C(u)\,du$, and, $\int_0^t C^R(u)\,du + C^R(t)/k_2^R$, are nearly noise-free. This means that almost all the errors in the LGA variables (in Eq. (1) in the main document) are due to $C(t)$, and therefore they will be nearly perfectly correlated, i.e., $r \approx 1$. Given this, according to [11], if the value of $\frac{\int_0^{t_i} C^R(u)\,du + C^R(t_i)/k_2^R}{C(t_i)}$ at point $\boldsymbol{H}$ has an error of ξ due to $C(t_i)$, then the value of $\frac{\int_0^{t_i} C(u)\,du}{C(t_i)}$ will have an error of an amount given by, $\left(\frac{\int_0^{t_i} C(u)\,du}{\int_0^{t_i} C^R(u)\,du + C^R(t_i)/k_2^R}\right) \cdot \xi$, due to $C(t_i)$. The slope of the straight line connecting the two points, $\boldsymbol{H}$ and $\boldsymbol{H}^*$, is therefore given by,

$$\begin{aligned}\frac{\Delta Y}{\Delta X} &= \left(\frac{\int_0^{t_i} C(u)\,du}{\int_0^{t_i} C^R(u)\,du + C^R(t_i)/k_2^R} \cdot \xi\right)/\xi \\ &= \frac{\int_0^{t_i} C(u)\,du}{\int_0^{t_i} C^R(u)\,du + C^R(t_i)/k_2^R}. \quad \text{(A3)}\end{aligned}$$

By Eqs. (A1) and (A2), the slope of the straight line between $\boldsymbol{H}$ and $\boldsymbol{H}^*$ is given by,

$$\begin{aligned}d_{i_{slope}} &= \frac{Y_i - y'_i}{X_i - x'_i} \\ &= \frac{w(X_i) - \alpha' r\sqrt{w(X_i)w(Y_i)}}{r\sqrt{w(X_i)w(Y_i)} - \alpha' w(Y_i)} \quad \text{(A4)}\end{aligned}$$

Since the errors in the LGA variables are highly correlated, we use the approximate, $r \approx 1$. Eq. (A4) thus becomes,

$$d_{i_{slope}} = \frac{w(X_i) - \alpha'\sqrt{w(X_i)w(Y_i)}}{\sqrt{w(X_i)w(Y_i)} - \alpha' w(Y_i)}, \quad \text{(A5)}$$

and by factorization it reduces to,

$$d_{i_{slope}} = \sqrt{\frac{w(X_i)}{w(Y_i)}}. \quad \text{(A6)}$$

Both Eqs. (A3) and (A6) refers to the slope of d_i. Together they give,

$$\begin{aligned}d_{i_{slope}} &= \sqrt{\frac{w(X_i)}{w(Y_i)}} \\ &= \frac{\int_0^{t_i} C(u)\,du}{\int_0^{t_i} C^R(u)\,du + C^R(t_i)/k_2^R}. \quad \text{(A7)}\end{aligned}$$

It is therefore that, a pair of $(w(X_i), w(Y_i))$ which satisfies Eq. (A7) would make appropriate weight functions for the LSC-based LGA.

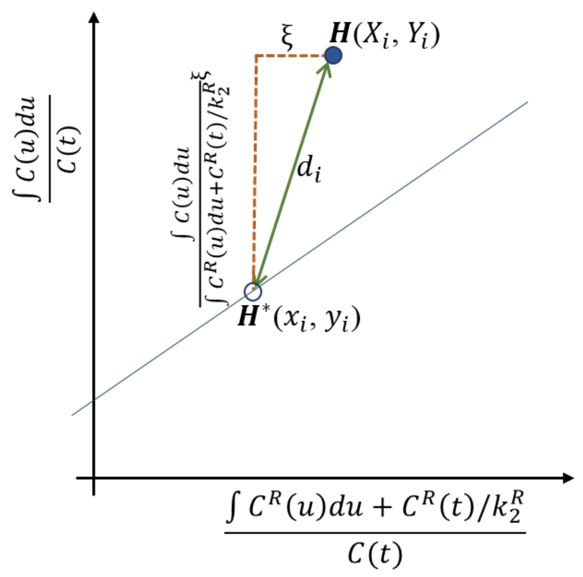

Figure S1: A schematic diagram for LSC. $\boldsymbol{H}$ is the *ith* observed noisy data point, whilst $\boldsymbol{H}^*$ is the corresponding estimated point lying on the estimated straight line. The point is that, for LSC, the directions of the residual vectors are not restricted in a single direction (*OLS residuals are restricted in the vertical direction*), but each residual points in a unique direction with an inclination described in Eqs. (A3 – A7).

The weight functions, $w(X_i) = 1/X_i^2$ and $w(Y_i) = 1/Y_i^2$, used in this study satisfy Eq. (A7) as,

$$\begin{aligned}\sqrt{\frac{w(X_i)}{w(Y_i)}} &= \sqrt{\frac{1/X_i^2}{1/Y_i^2}} = \frac{Y_i}{X_i} \\ &= \frac{\int_0^{t_i} C(u)du/C(t_i)}{\left(\int_0^{t_i} C^R(u)\, du + C^R(t_i)/k_2^R\right)/C(t_i)} \\ &= \frac{\int_0^{t_i} C(u)du}{\int_0^{t_i} C^R(u)\, du + C^R(t_i)/k_2^R},\end{aligned} \quad \text{(A8)}$$

making them appropriate for the LSC-based LGA.
