# Supplementary material for: A strategy to account for noise in the X-variable to reduce underestimation in Logan graphical analysis for quantifying receptor density in positron emission tomography
Source: BMC Med Imaging. 2020 Feb 10;20:15. doi: 10.1186/s12880-020-0421-6 (PMC7011280; doi:10.1186/s12880-020-0421-6)
Supplement: Supplementary file 2 — Additional file 2 tTACs and the corresponding Logan plot. The upper panel shows the mean tTACs of the region of interest (BPND=3.00) and the reference region. The lower panel shows the corresponding Logan plot. By visual observation of where the data points in the Logan plot attain a linear relationship, the point corresponding to Time=30 minutes was chosen; hence, t∗=30 min was used in this study. The data points (in the lower panel) used for BPND estimation are dotted in the middle. [file 12880_2020_421_MOESM2_ESM.pdf]

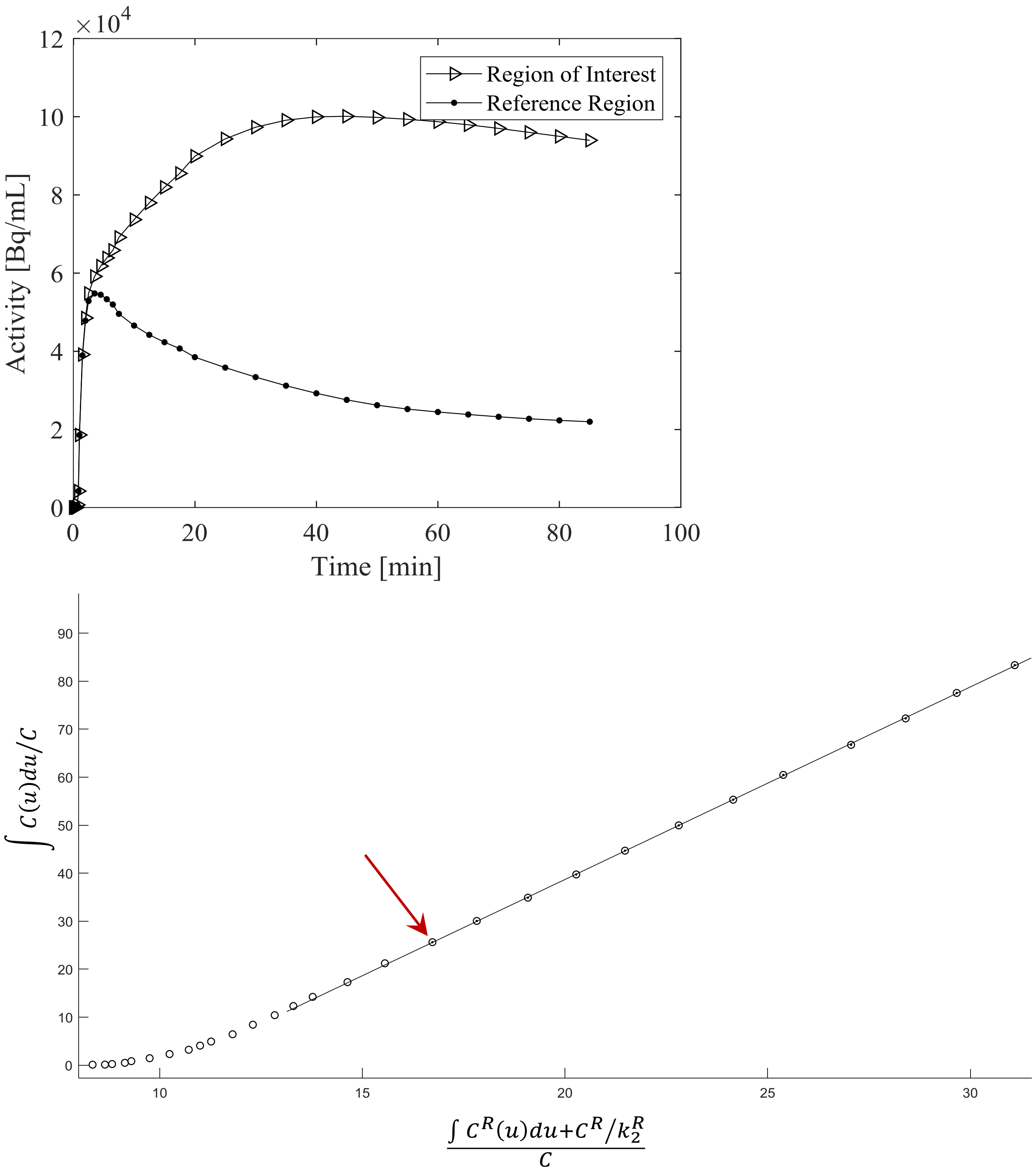

Figure S2: tTACs and the corresponding Logan plot. The upper panel consists of the mean (N = 1024) time-activity curves of the region of interest (BP_{ND} = 3.00) and the reference region. The lower panel shows the corresponding Logan plot obtained using the curves in the upper panel. By visual observation of where the data points in the lower panel attains a linear relationship, the 12th data point (indicated by the red arrow) from the right hand side was chosen. The corresponding data points in the upper panel are at $Time$ = 30 minutes. And thus t^* = 30 minutes was used for all methods in this study.
