# Supplementary material for: A strategy to account for noise in the X-variable to reduce underestimation in Logan graphical analysis for quantifying receptor density in positron emission tomography
Source: BMC Med Imaging. 2020 Feb 10;20:15. doi: 10.1186/s12880-020-0421-6 (PMC7011280; doi:10.1186/s12880-020-0421-6)
Supplement: Supplementary file 3 — Additional file 3 Comparisons of the distributions of the errors. This figure shows the distributions of the errors estimated by LSC (upper panel) and those estimated by OLS (lower panel). [file 12880_2020_421_MOESM3_ESM.pdf]

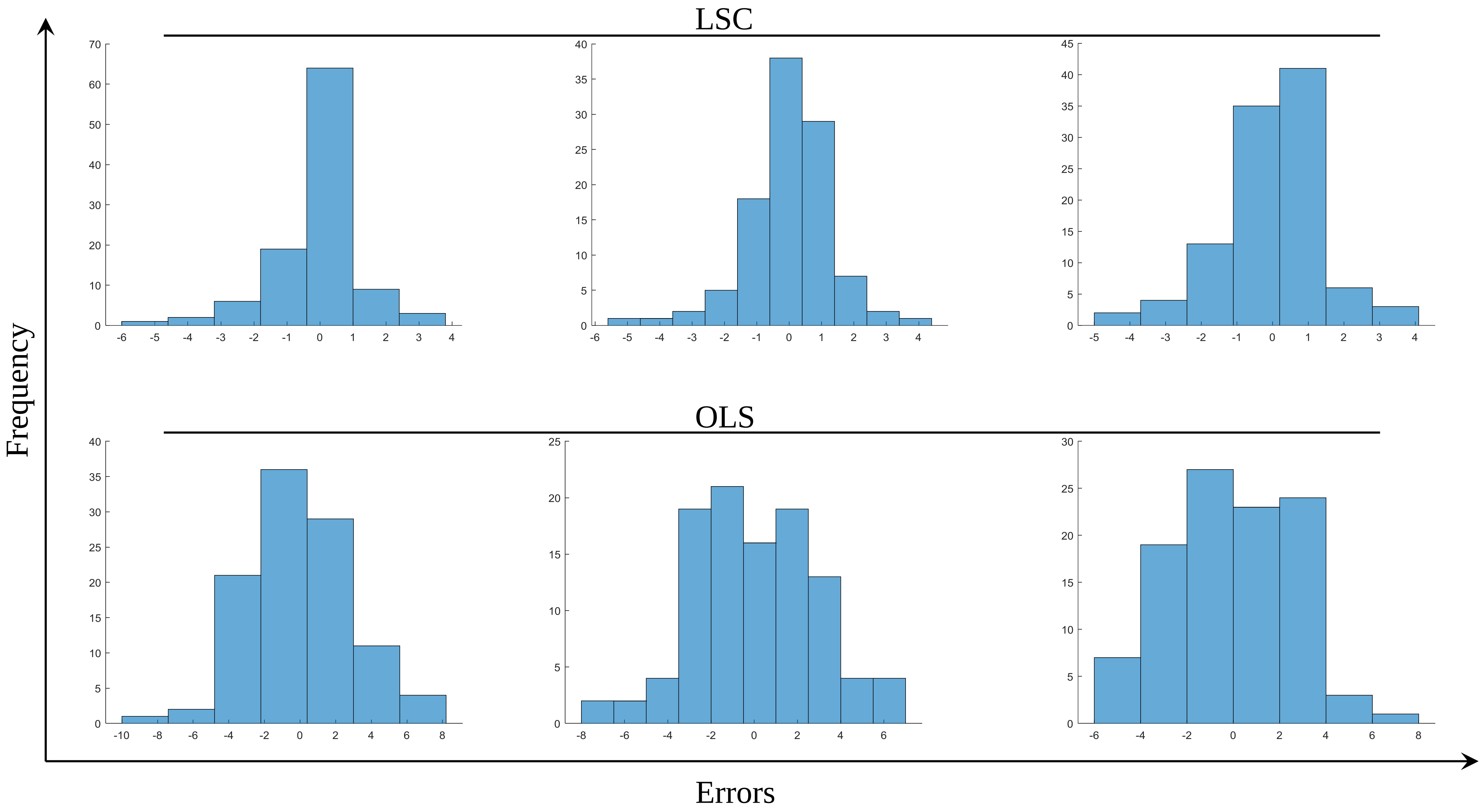

Figure S3: Comparison of the distributions of the LSC errors against the OLS errors. The upper panel shows the distributions of the errors estimated by LSC, and the lower panel shows those of OLS.
